# Designing implementation strategies for improving infection prevention and control in acute healthcare facilities in Malawi: A formative study protocol

**DOI:** 10.12688/wellcomeopenres.24040.2

**Published:** 2025-11-27

**Authors:** Dorica Ng'ambi, Tara Tancred, Nicholas Feasey, Wilned Zoto Hara, Owen Musopole, Thomasena O'Byrne

**Affiliations:** 1 Infection Biology - BDRI, Malawi-Liverpool-Wellcome Trust Clinical Research Programme, Blantyre, Southern Region, Malawi; 2 Clinical Sciences, Liverpool School of Tropical Medicine, Liverpool, England, UK; 3 The School of Medicine, University of St Andrews Faculty of Medicine, St Andrews, Scotland, UK; 4 Quality Improvement Unit, Queen Elizabeth Central Hospital, Blantyre, Southern Region, Malawi; 5 Quality Management Directorate, Government of Malawi Ministry of Health, Lilongwe, Central Region, Malawi

**Keywords:** Infection prevention and control; healthcare associated infections; formative research; co-design; implementation strategies; WHO core components of IPC

## Abstract

**Background:**

Healthcare associated infections (HAIs) are infections that patients acquire while receiving treatment and are not present during admission. The prevalence of HAIs is typically higher (15%) in low-and middle-income countries than that in high-income countries (7%). HAIs present a significant burden on patients, families, and health systems as they contribute to longer hospital stays, increased healthcare costs, and antimicrobial resistance. HAIs can be prevented or reduced by implementing infection prevention and control (IPC) measures. However, IPC measures are often poorly implemented due to resource shortages, lack of training, and other systemic challenges. The goals of this formative study were twofold: 1. to carry out a situational analysis of IPC practices for HAI control in three hospitals in Southern Malawi, highlighting specific bottlenecks and enablers of IPC practices; and 2. to co-design tailored implementation strategies based on insights from situational analysis using participatory approaches with key IPC stakeholders to support more consistent and effective IPC implementation at the study sites.

**Methods:**

The study will be conducted in three health facilities in Malawi representing different healthcare levels: Queen Elizabeth Central Hospital, Zomba Central Hospital, and Chikwawa District Hospital. For situational analysis, six data collection tools will be used: a desk review of IPC policies and guidelines, the World Health Organization (WHO) IPC Assessment Framework, participant and non-participant structured observations, interviews, and focus group discussions. The participatory component involves a three-day co-design workshop. Participants in both study components will include healthcare workers, support staff, policymakers, patients, and patient caregivers (guardians). Descriptive statistics will be used to analyse the quantitative data. A thematic framework analysis using NVivo 12 will be done on the qualitative data. The findings will be disseminated through workshops, academic publications, and stakeholder meetings.

**Conclusion:**

Multifaceted IPC implementation strategies tailored to the context of each hospital will be designed.

## Plain language summary

Healthcare associated infections (HAIs) are infections that patients catch while being treated in health facilities. These infections are a significant health problem, especially in low-resource settings, such as Malawi. HAIs can lead to longer hospital stays, cost more money for patients and hospitals, and make it more difficult to treat illnesses and diseases because of drug resistance. Common types of hospital acquired infections include infections after surgery, infections from cannulas and catheters, and pneumonia acquired in hospitals.

The World Health Organization (WHO) has established guidelines on infection prevention and control (IPC) to help hospitals prevent these infections. However, many hospitals in Malawi struggle to follow these guidelines because of challenges such as lack of staff, lack of training, and shortage of necessary supplies.

This study focused on developing context-specific practical ways to improve IPC practices in hospitals in Malawi.

This study will be conducted in three hospitals in Malawi. We aim to: 1. understand how IPC is currently being implemented in hospitals; 2. identify what makes it difficult or easy to follow IPC practices; and 3. involve everyone in creating a plan to improve the IPC. To do so, we will collect data from healthcare workers, policymakers, patients, caregivers, and guardians. We will interview some of these participants and conduct group discussions with others. We will also conduct observations of IPC practices. After learning from the collected data, we will work with some of these participants to develop practical ways to improve IPC practices in these hospitals.

Findings from this research will be shared with hospital staff, the hospital community (patients, guardians, caregivers, and visitors), the government, and the public to promote better IPC practices across the country.

## Introduction

Healthcare associated infections (HAIs) are infections that patients contract while receiving care and treatment for other illnesses in healthcare facilities ^1, 2^. The World Health Organization (WHO), has estimated the prevalence of HAIs is estimated at 7% in developed countries and 15% in developing countries ^2– 5^. The most common HAIs are surgical site infections (SSIs), catheter-associated urinary tract infections (CAUTIs), central line-associated bloodstream infections (CLABSIs) and hospital acquired pneumonia (HAP). HAIs present a huge burden on patients, guardians, health facilities, and the country’s health system. Patients with HAI tend to have longer and costlier hospital stays. Treatments tend to be less effective and promote antimicrobial resistance (AMR). HAIs result from a complex interplay between insufficient policies, infrastructure, and capacities of health workers and auxiliary staff, as well as poor patient and caregiver/guardian-related practices ^3, 4, 6^. HAIs are highly preventable through the implementation of guidelines on infection prevention and control (IPC). IPC practices are evidence-based approaches that prevent health workers, guardians/visitors, and patients from acquiring avoidable infections during the provision of healthcare services ^1, 6, 7^.

SSIs are postoperative infections that occur at the surgical site and are among the most common HAIs worldwide ^8, 9^. A study including seven African countries found an overall SSI prevalence of 22%; specifically in Malawi, the prevalence was 22% ^9, 10^. CLABSIs and CAUTIs are device-associated infections that occur after 48 hours or more of having a device inserted, either a central line/peripheral cannula or urinary catheter ^11– 13^. The prevalence of CLABSI and CAUTI is 20–21% in Africa ^5^. HAP is a pneumonia that occurs 48 hours or more after admission to the hospital and is not present at the time of admission ^14, 15^.

The WHO has developed guidelines for the implementation of IPC and prevention of HAIs, based on eight core components that are considered essential to ensuring patient safety and improving quality of care, including: 1) IPC programming; 2) IPC guidelines; 3) IPC education and training; 4) HAI surveillance; 5) multimodal improvement strategy; 6) monitoring/audit and feedback of IPC activities; 7) workload, staffing, and bed occupancy; and 8) built environment, materials, and equipment for IPC ^16^. It is recommended that the implementation of the core components of IPC should be at both the national and facility levels; however, there is limited tailored guidance for what this means, in practice, at each level.

These core components complement each other. For example, it is suggested that an active IPC program will provide a plan for how other core components can be implemented to improve IPC practices and reduce HAIs. However, for an IPC program to exist, there should also be an enabling environment, reflected in two of the core components (workload, staffing and bed occupancy, and built environment, materials, and equipment). The use of the multimodal improvement strategy (which encompasses system change and infrastructure improvement, education and training of healthcare workers and key players, monitoring and feedback, reminders in the workplace/communications, and culture change, especially in leadership) suggests that a bundle of approaches should be used in conjunction to facilitate optimal implementation.

There are many further challenges to the implementation of the WHO core components of IPC, including shortages of staff (for example, in Malawi, one nurse provides care to 30–80 inpatients per shift, which compromises the care given to every inpatient) ^17^, inadequate IPC training of health care workers (HCWs) and patients/patient guardians, and a lack of IPC resources and supplies (i.e., alcohol-based hand rub, soap, and clean running water). Systemic challenges are often encountered in the short-term solutions. For example, as a tentative solution to the shortage of nursing staff, in many African regions, health facilities depend on patient guardians/caregivers to supplement the overburdened work force and are directly involved in the patient’s activities of daily living, including bathing, feeding, cooking, transporting them, and assisting with rehabilitation exercises ^18, 19^. A guardian can be a patient’s relative, friend, or someone who has been assigned to care for the patient during hospitalization by the patient or the patient’s family. Therefore, it is important that guardians be considered and included in the IPC program. Such inclusion could involve providing IPC-related health education for individuals to know what is expected when offering care to patients to prevent avoidable infections ^17– 19^.

In Malawi, IPC guidelines have been adapted from a combination of the WHO, Centre for Disease Control (CDC), and Infection Control Africa Network (ICAN) guidelines; however, there is no context-specific and locally adapted plan for implementing IPC guidelines on the core components. Well-developed or adapted guidelines without formal support for their implementation are of limited value ^15, 20^. While the WHO has further developed some implementation guidelines for WHO IPC minimum core component requirements ^21^, there are still gaps in moving this guidance into practice, and adherence to WHO recommendations remains a critical challenge. These gaps were evident in the 2019 Global Survey on Infection Prevention and Control reports. LMICs scored at a “basic” level of IPC implementation on average, while notably having limited implementation of IPC guidelines, training and education, monitoring, audit, feedback, and HAI surveillance ^22^. This raises the question of what factors lead to low adherence to the WHO recommendations in healthcare facilities and what can be done to improve IPC practices and reduce HAIs. There is a dearth of research on IPC implementation, particularly in LMIC settings, to help explore how patient safety and quality of care can be improved. In Malawi, despite the availability of national IPC guidelines, there are IPC implementation challenges. These include lack of operationalisation plans, inconsistent adherence to IPC practices, shortage of IPC resources and lack of training for staff. Given these gaps, this study aims to co-develop locally adapted implementation strategies—interventions that are specifically in place to support the implementation of evidence-based practices—to support the routine use of the WHO Core Components of IPC at the facility level in Malawi.

### Specific study objectives

1. To understand the current landscape of IPC in Malawi, including three acute healthcare facilities, using a mixed-methods situational analysis.
2. To identify potential barriers and enablers to the implementation of the WHO Core Components of IPC at the facility level in Malawi.
3. To use participatory methods, co-design with IPC stakeholder context-specific strategies to support the implementation of the WHO Core Components of IPC for use within acute healthcare facilities in Malawi.

## Methods

### Study design

This formative study will have two components. The first is a mixed-methods situational analysis, drawing extensively on qualitative approaches to understand how IPC is currently being conducted at our three study sites. This situational analysis will also include the exploration of potential barriers and enablers of IPC practices. Second, we will conduct a participatory workshop in which key findings from the situational analysis will be shared, after which we will co-design implementation strategies with participating stakeholders to support and improve IPC practices. In line with the best practice guidance for designing and evaluating complex interventions ^23^, in addition to designing context-appropriate implementation strategies, this formative phase will further provide a basis for building a relational foundation with stakeholders to support the use and evaluation of these implementation strategies in a subsequent study.

### Study period

Primary data collection will take place over a period of one year, from April 2023 to March 2024, in Blantyre, Chikwawa, and Zomba.

### Study sites

Primary data will be collected from the medical and surgical wards in three health facilities in Malawi: Queen Elizabeth Central Hospital (QECH), Zomba Central Hospital (ZCH) and Chikwawa District Hospital (CDH). These hospitals were purposively selected as they represent secondary and tertiary levels of healthcare facilities, and are teaching, central, and district hospitals, respectively.

QECH provides specialized medical care, and it is the largest urban government teaching and tertiary level hospital in Blantyre, which has a capacity of approximately 1350 beds, but frequently operates above its capacity ^24^. Located in the Southern region of Malawi, ZCH is a tertiary hospital, although it also works as a secondary level hospital for the Zomba district. ZCH has a capacity of 620 beds but operates above its capacity. The CDH is a secondary level hospital with a capacity of 250 beds and is situated in the Chikwawa District in southern Malawi.

### Study populations

The study population will include healthcare workers, housekeeping staff, patients, and guardians responsible for carrying out IPC practices in medical and surgical wards, and the leadership/managerial staff who support IPC practices at each site. We will also include public health practitioners, researchers, and other stakeholders from the Ministry of Health (MoH), especially from the Quality Management Directorate (QMD), as key policy holders for IPC implementation, non-governmental organizations, and funding bodies involved in IPC.

### Sample size

We estimate that 200 people will be recruited for this study. As indicated in Table 1, in the situational analysis, we anticipate including 40 participants in interviews (30 in-depth interviews with health workers and auxiliary staff, 10 key informant interviews), 60 participants across six focus group discussions (FGDs), and 60 participants in observation. In the second (participatory) phase of the study, we selected 30 participants from the same group who participated in the interviews, observations, and FGDs. The sample sizes in this study are consistent with recommendations in qualitative research. For interviews, data saturation is usually reached with 12–30 interviews. Therefore, interviewing 30 staff members will be sufficient to reach saturation. For FGDs a size of 6–10 participants is recommended to facilitate meaningful interaction and gain insights. We anticipate that observation of 60 HCWs across the three facilities conducting IPC practices will give a good representation of how these IPC practices are done. Participants will be identified using a combination of purposive and snowball sampling techniques. To support maximum variation sampling, we will purposefully select participants from a wide range of backgrounds, job roles, experiences, and characteristics relevant to this study. These sampling techniques are appropriate given that the aim is to enrol stakeholders involved with or who have experience in IPC. Data collection will occur until theoretical saturation is reached. Saturation will be defined as the point at which no new insights are being found.

**Table 1.** Sample size.

Data collection method	Type of participants	No. participants per facility	Total number of participants
*Situational analysis*			
Participant Observation	Healthcare workers	20	60
Interviews	Healthcare workers	10	30
	IPC stakeholders external to the hospital facilities		10
Focus group discussions *	Cleaners	10	30
	Guardians	10	30
*Participatory research*			
Implementation strategy co-design workshop	Study team	10	42
	National IPC focal persons	2	
	Infection prevention and control Association - Malawi	1	
	Public health Institute of Malawi	1	
	ZCH HCWs **	10	
	QECH HCWs **	10	
	Cleaners	4	
	Patient guardians	4	

*Six to be held in total, one with cleaners and one with guardians per facility, each with approximately 10 participants. ** the group of HCWs will include the representatives from the study wards, facility IPC focal person, quality improvement focal person and management

### Data collection

We will use five different data collection approaches within the situational analysis: desk/document review, Infection Prevention and Control Assessment Framework (IPCAF) survey, participant and non-participant observation, interviews (semi-structured in-depth interviews and key informant interviews), and focus group discussions. We will use one method (a participatory workshop) in the second part of the study, we will conduct a participatory workshop. A desk review tool will be used to assess the content of the documents and how it aligns with the national guidelines. IPCAF survey will give scores across the eight core components. The observation tool will assess the indication, moment, cadre and specific aseptic techniques during invasive procedures. While the interviews and FGD guides will generate data on knowledge, experiences, barriers/facilitators and perceptions of staff.

### Situational analysis

***Desk/Document review***

We will conduct an extensive desk/documentary review to identify and review existing IPC guidelines, policies, standard operating procedures, and protocols for the prevention of HAI at the facility and national levels. Grey literature on IPC will be obtained from the QECH, ZCH, CDH, and Malawi MoH archives. If archival material is not accessible, we will ask permission to access the material from the relevant authorizing personnel and seek consent, where necessary, for its use as formal research data. To guide desk review and document analysis, a tool was developed Https://doi.org/10.5281/zenodo.15202999

***Infection Prevention and Control Assessment Framework Survey***

At each study site, an electronic survey will be conducted in the surgical and medical departments using the WHO IPCAF tool which is freely available for use by health facilities Https://doi.org/10.5281/zenodo.15202999. The IPCAF is a self-administered, structured tool that will be conducted by the facility IPC focal person with the support of the study team. The IPCAF evaluates the current IPC situation in each facility, including key components such as leadership, education, surveillance, multimodal strategies, and scoring facilities across different levels of IPC maturity (Inadequate, Basic, Intermediate, and Advanced). For example, under Core Component 2: Education and Training, a hospital may score at the intermediate level if it provides periodic IPC training but lacks a structured, mandatory curriculum. Similarly, in Core Component 5: Surveillance, a facility may be rated basic if it collects infection data but does not yet use it for active outbreak response and prevention strategies. The study implementation lead (DN) will facilitate contact with hospital directors from each health facility for permission to use the tool and arrange training on the IPCAF tool. Thereafter, an appropriate date and time will be determined for the assessment. The tool has indicators drawn from WHO guidelines on the eight core components of IPC (100 points per component for a total possible score of 800). The scores were divided into four levels ( Table 2). Overall, facility IPCAF scores can be used as indicators of IPC implementation level. The data from the IPCAF tool will also inform the data collection instruments for interviews, FGDs, and observations.

**Table 2.** IPCAF scoring interpretation.

Score	Level	Interpretation
0 – 200	Inadequate	IPC CC implementation is deficient. Significant improvement is required.
201 – 400	Basic	Some aspects of the IPC CCs are in place, but not sufficiently implemented. Further improvement is required.
401 – 600	Intermediate	Most aspects of IPC CC are appropriately implemented. Continue to improve the scope and quality of implementation and focus on the development of long-term plans to sustain and further promote the existing IPC program.
601 – 800	Advanced	The IPC CCs are fully implemented according to the WHO recommendations and appropriate to the needs of your facility

### Observation

Observations of IPC practices will be conducted to produce data on what people do (as opposed to what they say they do). During periods of participant (of people) and non-participant observation (of environments and infrastructure), we are interested in understanding IPC practices, especially those for preventing HAI embedded within daily work routines and interactions between persons.

***Participant observation***

Observations of healthcare workers will be conducted in medical and surgical units across the three sites. We will use a tool which we have developed after review of literature from the CDC, MoH IPC guidelines and assessment tools, Infection Control Africa Network and WHO Https://doi.org/10.5281/zenodo.15202999. Indicators are subdivided into IPC practices to be followed when performing urinary catheterization, intravenous cannulation, or wound dressing procedures. This tool will be used to observe 60 HCWs across the three study sites, divided equally across male and female units in each of the surgical and medical units. Individual HCWs will be observed by performing the procedures on five different patients in a specific period. The observations will be conducted by research assistants with IPC knowledge and skills. Data from participant observation will be collected by the observer using a standardized online form built into the Research Data Capture (RedCap).

***Non-participant observation***

Given our wide interest in HAIs and the IPC landscape and environment, our attention will not only be on staff, patients, and guardians, but also on daily activities in the setting. Non-participant observation (that is, observing the setting versus observing the behaviours of specific individuals) will be conducted to assist in describing what is happening on the ground versus what people/documents say is happening. Trained members of the study team will spend time in the medical and surgical units observing daily practices, interactions, flow of work, and life within the departments/units across the three sites. The study team will record their observations in field notes.

### Interviews

***Semi-structured interviews***

We will conduct semi-structured interviews with HCWs to establish their knowledge of IPC, the current IPC landscape within the study sites, and their implementation of IPC core components. We aim to recruit 10 HCWs per facility (30 HCWs across the study sites), including individuals working within the medical and surgical departments, IPC focal people, and some members of department management.

The HCWs will be briefed on the study and study objectives by the study team during a convenient time (e.g., before the morning handover and lunchtimes). After the briefing, information leaflets will be distributed to prospective participants, who will be given 24 h to read through the leaflet and determine whether they would like to participate. The study team will revisit the individual departments/units after at least 24 hours to ask if any individuals are interested in participating.

Interviews with participants will be conducted in both Chichewa (a local language) and English by research assistants within office/hospital spaces, or if this is contextually inappropriate (for instance, if no private space is available), an alternative location will be selected. They are expected to take 30–60 minutes.

***Key informant interviews***

Key informant interviews will be conducted to understand the upstream factors that shape IPC practices in Malawi, including research related to HAIs. Key informant interview guides will be informed by the existing academic and grey literature on IPC from the desk/document review and observational data. We will recruit 20 IPC stakeholders, including people from the three healthcare facilities, the QMD, and IPC government partners (WHO, Public Health Institute of Malawi, Infection Prevention and Control Association of Malawi). The study information leaflets will be sent to prospective key informants either via email or delivered by the study team in person to offices/places of work. The study team will send a follow-up email, make a phone call, or visit prospective IPC stakeholder participants to ask if any individuals are interested in participating. Ideally, face-to-face interviews will be conducted; however, online (on Zoom or Teams) or telephone will be offered if necessary. They are expected to take 60–90 min.

### Focus Group Discussions

We will conduct focus group discussions (FGDs) with (i) guardians/caretakers of patients within the medical and surgical units and (ii) housekeeping staff working within the sites. The purpose of conducting guardian FGDs is to understand their experiences with IPC while they have been at the hospital, including how they think IPC may improve. FGDs with cleaners will explore their perspectives on IPC, the role that they play in IPC/health systems/hospital teams, and their understanding of any IPC training they have received. We aim to conduct six FGDs in total (one with guardians and one with housekeeping staff at each site). Based on the size of the medical and surgical units across the facilities and with the desire to cause minimal disruption, we believe that two FGDs per facility will be sufficient for this formative research. We intend to consent 10 participants per FGD.

Guardians who have stayed in the study units for more than two days will be purposively identified in consultation with the healthcare staff. These guardians will be selected from different wards and divided into groups of males and females. We will consult with the ward management to ensure that the guardians do not have patients who are critically ill and are therefore available to attend an FGD discussion. The study team will approach the housekeeping staff and ask them if they wish to participate in the study. Additionally, a leaflet about FGDs will be posted on hospital noticeboards with the study team contact details provided. Prospective participants for all FGDs will be given a study information leaflet in the local language and at least 24 h to consider their participation in the study Https://doi.org/10.5281/zenodo.15202999 The study team will revisit prospective participants after a minimum of 24 h to ask if they are interested in participating in the study. The study leaflet and consent form will be read out for prospective participants with literacy limitations. An impartial witness will be used to ensure that participant understand the study and consent form. This impartial witness will sign the witness section on the consent form, and the participant will use his/her thumb print on the consent form.

Each FGD will last between 60–90 minutes and will take place in a quiet location (such as a meeting area inside or outside within the grounds of the hospital). Facilitators and note-takers will be present.

### Participatory component

***Stakeholder consultation and design workshop***

Co-designing of the implementation strategies will involve a workshop with key stakeholders (including research staff, facility-based healthcare workers and auxiliary staff, IPC key informants, and community members/guardians). Participants will be identified during situational analysis. The workshop will be conducted over a three-day period and will include approximately 30 people. During the workshop, the study team will present key findings from the situational analysis. We will use group exercises/discussions to reflect on these findings to co-design context-specific implementation strategies to improve the routine use of IPC Core Components in each study site.

Data will be collected during workshops and meetings through observations and note-taking of interactions and dynamics between participants. Photographs of the process will also be taken.

### Data analysis

Descriptive statistics and framework analysis will be used to identify patterns and key insights relevant to the study’s objectives.

***Desk review***

The selected documents will be appraised against the WHO core component guidance to identify the gaps and strengths that exist in the guidelines/IPC documents at the facility level. We will then compare and aggregate these findings with data from other sources.

***IPCAF survey and participant observation***

The IPCAF data will be analysed in Microsoft Excel using descriptive statistics. The IPCAF scores will be summarized using median and interquartile ranges. The mean scores will also be calculated. Data from the HCWs’ observation checklists will be analysed in Excel and presented as data summaries, reports, and graphs.

***Interviews and FGDs***

Qualitative data in the form of field notes, interviews, and FGD transcripts will be uploaded into the Lumivero (2024) NVivo (Version 12). Lumivero. https://www.lumivero.com/ for coding. Recorded data will be transcribed and translated. Anonymized transcripts will be analysed using a combined deductive framework analysis, followed by an inductive thematic analysis within each component of the framework. As the coding advances within each framework component, higher order codes will be developed to explain and theorize the patterns and themes in the data. Coding reliability will be checked by double coding 20% of the transcript noting coder agreement and resolving disagreements. Findings and interpretations, including transcripts, fieldnote summaries, and written analyses, will be discussed with the wider IPC-Implement research team to maintain consistency in coding and depth of analysis.

### Cross-site analysis

The study team will conduct regular reflection meetings to discuss emerging data as the research progresses, inviting the MoH to join these reflections when available. Using framework analysis, data from the three sites will be analysed as separate project files in NVivo 12. By comparing the analyses, we aim to identify common themes and connections between sites, as well as points of departure and differences. Member checking of the qualitative data will be done with selected HCWs, guardians and housekeeping staff. Key findings from this study will be presented to participants in the participatory component of this study for discussion and co-development of a multimodal strategy for improving IPC practices in the CDH, ZCH, and QECH.

### Data management

Data will be managed through infrastructure set up within the Malawi-Liverpool-Wellcome Research Programme.

Observational data and IPCAF survey data will be stored on the MLW Research Electronic Data Capture (REDCap) server, anonymized, and transferred to a Microsoft Excel spreadsheet for analysis.

Data to be uploaded to REDCap will be collected using tablets and will include interview recordings, transcripts, field notes, data on IPCAF, and observations. The data will be backed up to the MLW’s secure cloud storage. The full anonymization process will occur following peer-reviewed publication, but all contact details collected from participants throughout the study will be destroyed at the end of the study follow-up for data cleaning and query resolution. Once transcripts have been generated and quality checked from the audio files, the researcher will delete the audio files.

All raw data will be stored in password-protected files/folders, and second-order summaries of field notes, interview transcripts, photos, and other field documents will be uploaded to a data repository (with participant consent). Details on how to access the data will be published with each study publication, and access will be granted based on a case-by-case request and approval.

The data and paper-based records of this project will be destroyed after 5 years. All devices and paper-based tools containing data will be securely stored in offices at the Malawi Liverpool Wellcome Research Programme (MLW) data unit and in a locked data repository room for long-term storage. The MLW Data Office will perform daily data backups, with an offsite backup done weekly.

## Ethics and consent

Ethical approvals to conduct the study were obtained from the Kamuzu University of Health Sciences Research Ethics Committee (COMREC P.02/23/3993 on 17 ^th^ May 2023) and the Liverpool School of Tropical Medicine Research Ethics Committee (LSTM REC - 23-007 on 2 ^nd^ June 2023). Before commencing research activities, we will approach relevant political and social institutions to explain the objectives of the study and seek permission to work in the proposed areas. This study will adhere to the Declaration of Helsinki.

At the time of enrolment, written informed consent forms and information sheets on study participation will be provided for each adult participant (over the age of majority according to local regulations in Malawi (, that i.e.is, 18 years of age). Where applicable, the consent forms will include consent for audio recordings, the use of direct quotes, and photography (within the participatory workshop only), which participants may opt out of but still maintain their participation. Participants will be given at least 24 hours to consider their participation between when they are provided with the study information and when they are consented.

The formative research will be observational and thus is not expected to pose harm to participants, barring interruptions, or minor inconveniences from participating in research activities. However, we will only conduct research activities at appropriate times as directed by participants. We are conscious that we may take up people’s time and will therefore ensure that we arrange data collection at times suitable to the participant, respecting when there may be cancellations or postponements. Participants travelling to workshops will be reimbursed for their transport and accommodation.

During the research process, it is possible to uncover poor IPC practices and/or poor clinical practices that need to be resolved. During such times, we will ensure that such issues are first discussed with the principal investigator and then with the relevant individuals in and heads of the department. For those being observed, it is possible for individuals to feel scrutinized or uncomfortable. We will stress that they will not be identifiable from any notes that we have taken. If individuals voice concerns about being observed, they will not be observed.

The in-depth nature of data collection methods, particularly participant observation, safeguarding participants ’privacy is a priority. We will implement strict measures to ensure their privacy is respected. All interviews, and FGDs will be conducted in a private space where conversations cannot be overheard. Where participant observation takes place in public spaces, it will be made very clear to participants at the outset that they can make informed choices as to when to share information. Study researchers will ensure that participants are aware that only the research team will have access to raw data (including both raw data and data uploaded to REDCap). Any data shared beyond the group will be subject to approval by the study team on a case-by-case basis.

We will ensure that participants fully understand their participation in the study is voluntary and that they will not face any consequences should they choose not to respond to some questions, refuse to participate, or withdraw from the study at any point. More broadly, we will ensure that participants understand that this study aims to contribute to improved policies and guidelines for IPC that will benefit people in Malawi.

All participants will be assigned pseudonyms in publications and other research outputs from this study. In the case of public health practitioners, policymakers, and other stakeholders—individuals with more unique and potentially identifiable roles—it will be made clear during the consent process that there may be limits to their anonymity in research output. We will also offer them the option of not using direct quotations from the interviews.

## Dissemination of the results

Dissemination of situational analysis is, in part, built within this study through a participatory workshop. However, participants who are not part of the workshop will have their findings shared through feedback workshops with staff working in the medical and surgical departments which be held immediately after situational analysis. After the participatory workshop we will then hold wider dissemination meetings of study findings with the CDH, QECH, and ZCH senior management teams. The results will also be presented through an ongoing dialogue with the QMD technical working group, including QMD zonal officers. We will also share our findings with the Kamuzu University of Health Sciences Research Ethics Committee, including other academics, on their research dissemination day. The results from this study will be further disseminated to other stakeholders, including additional MoH Malawi representatives, COMREC, and LSTM REC. We will also present findings at national and international conferences and aim to publish them in academic journals.

## Expected findings

The key outputs from this work will provide a comprehensive understanding of IPC practices in three hospitals in southern Malawi, including an improved understanding of key bottlenecks and opportunities for strengthening IPC practices, as well as co-designed implementation strategies to enable routine use of the IPC Core Components. These findings will be reported in terms of baseline IPC practices compliance percentage, IPCAF scores and the number and type of co-develop implementation strategies. We will have insights into who will use and champion these implementation strategies, where and how they should be used, and for how long. We will then build on these outputs, supporting and evaluating these implementation strategies in a larger piece

## Data Availability

No data associated with this article. Data collection tools. Https://doi.org/10.5281/zenodo.15202999 ^25^ This project contains the following extended data: Document review guide Infection prevention and control assessment framework tool Healthcare observation tool Participant information leaflet for patient guardians and cleaning staff Data available under CC By 4.0 Creative Commons Attribution 4.0 International
