# Designing implementation strategies for improving infection prevention and control in acute healthcare facilities in Malawi: A formative study protocol

**DOI:** 10.12688/wellcomeopenres.24040.4

**Published:** 2026-04-09

**Authors:** Dorica Ng'ambi, Tara Tancred, Nicholas Feasey, Wilned Zoto Hara, Owen Musopole, Thomasena O'Byrne

**Affiliations:** 1 Infection Biology - BDRI, Malawi-Liverpool-Wellcome Trust Clinical Research Programme, Blantyre, Southern Region, Malawi; 2 Clinical Sciences, Liverpool School of Tropical Medicine, Liverpool, England, UK; 3 The School of Medicine, University of St Andrews Faculty of Medicine, St Andrews, Scotland, UK; 4 Quality Improvement Unit, Queen Elizabeth Central Hospital, Blantyre, Southern Region, Malawi; 5 Quality Management Directorate, Government of Malawi Ministry of Health, Lilongwe, Central Region, Malawi

**Keywords:** Infection prevention and control; healthcare associated infections; formative research; co-design; implementation strategies; WHO core components of IPC

## Abstract

**Background:**

Healthcare associated infections (HAIs) are infections that patients acquire while receiving treatment and are not present during admission. The prevalence of HAIs is typically higher (15%) in low-and middle-income countries than that in high-income countries (7%). HAIs present a significant burden on patients, families, and health systems as they contribute to longer hospital stays, increased healthcare costs, and antimicrobial resistance. HAIs can be prevented or reduced by implementing infection prevention and control (IPC) measures. However, IPC measures are often poorly implemented due to resource shortages, lack of training, and other systemic challenges. The goals of this formative study were twofold: 1. to carry out a situational analysis of IPC practices for HAI control in three hospitals in Southern Malawi, highlighting specific bottlenecks and enablers of IPC practices; and 2. to co-design tailored implementation strategies based on insights from situational analysis using participatory approaches with key IPC stakeholders to support more consistent and effective IPC implementation at the study sites.

**Methods:**

The study will be conducted in three health facilities in Malawi representing different healthcare levels: Queen Elizabeth Central Hospital, Zomba Central Hospital, and Chikwawa District Hospital. For situational analysis, six data collection tools will be used: a desk review of IPC policies and guidelines, the World Health Organization IPC Assessment Framework, participant and non-participant structured observations, interviews, and focus group discussions. The participatory component involves a three-day co-design workshop. Participants in both study components will include healthcare workers, support staff, policymakers, patients, and patient caregivers (guardians). Descriptive statistics will be used to analyse the quantitative data. A thematic framework analysis using NVivo 12 will be done on the qualitative data. The findings will be disseminated through workshops, academic publications, and stakeholder meetings.

**Conclusion:**

Multifaceted IPC implementation strategies tailored to the context of each hospital will be designed.

## Plain language summary

Healthcare associated infections (HAIs) are infections that patients catch while being treated in health facilities. These infections are a significant health problem, especially in low-resource settings, such as Malawi. HAIs can lead to longer hospital stays, cost more money for patients and hospitals, and make it more difficult to treat illnesses and diseases because of drug resistance. Common types of hospital acquired infections include infections after surgery, infections from cannulas and catheters, and pneumonia acquired in hospitals.

The World Health Organization (WHO) has established guidelines on infection prevention and control (IPC) to help hospitals prevent these infections. However, many hospitals in Malawi struggle to follow these guidelines because of challenges such as lack of staff, lack of training, and shortage of necessary supplies.

This study focused on developing context-specific practical ways to improve IPC practices in hospitals in Malawi.

This study will be conducted in three hospitals in Malawi. We aim to: 1. understand how IPC is currently being implemented in hospitals; 2. identify what makes it difficult or easy to follow IPC practices; and 3. involve everyone in creating a plan to improve the IPC. To do so, we will collect data from healthcare workers, policymakers, patients, caregivers, and guardians. We will interview some of these participants and conduct group discussions with others. We will also conduct observations of IPC practices. After learning from the collected data, we will work with some of these participants to develop practical ways to improve IPC practices in these hospitals.

Findings from this research will be shared with hospital staff, the hospital community (patients, guardians, caregivers, and visitors), the government, and the public to promote better IPC practices across the country.

## Introduction

Healthcare associated infections (HAIs) are infections that patients contract while receiving care and treatment for other illnesses in healthcare facilities. ^1^ ^,^ ^2^ The World Health Organization (WHO) has estimated the prevalence of HAIs at 7% in high-income countries and 15% in low-income countries. ^2^ ^–^ ^5^ The most common HAIs are surgical site infections (SSIs), catheter-associated urinary tract infections (CAUTIs), central line-associated bloodstream infections (CLABSIs) and hospital acquired pneumonia. HAIs present a huge burden on patients, guardians, health facilities, and the country’s health system. Patients with HAI tend to have longer and costlier hospital stays. Treatments tend to be less effective and promote antimicrobial resistance (AMR). HAIs result from a complex interplay between insufficient policies, infrastructure, and capacities of health workers and auxiliary staff, as well as poor patient and caregiver/guardian-related practices. ^3^ ^,^ ^4^ ^,^ ^6^ HAIs are highly preventable through the implementation of infection prevention and control (IPC) practices. IPC practices are evidence-based approaches that prevent health workers, guardians/visitors, and patients from acquiring avoidable infections during the provision of healthcare services. ^1^ ^,^ ^6^ ^,^ ^7^

SSIs are postoperative infections that occur at the surgical site and are among the most common HAIs worldwide. ^5^ ^,^ ^8^ A study including seven African countries found an overall SSI prevalence of 22%; specifically in Malawi, the prevalence was also 22%. ^8^ ^,^ ^9^ CLABSIs and CAUTIs are device-associated infections that occur after 48 hours or more of having a device inserted, either a central line/peripheral cannula or urinary catheter. ^10^ ^–^ ^13^ The prevalence of CLABSI and CAUTI is 20–21% in Africa. ^5^ Hospital acquired pneumonia occurs 48 hours or more after admission to the hospital and is not present at the time of admission. ^11^ ^,^ ^12^ ^,^ ^14^

The WHO has developed guidelines for the implementation of IPC practices and prevention of HAIs based on eight core components that are considered essential to ensuring patient safety and improving quality of care, including: 1) IPC programming; 2) IPC guidelines; 3) IPC education and training; 4) HAI surveillance; 5) a multimodal improvement strategy; 6) monitoring/audit and feedback of IPC activities; 7) workload, staffing, and bed occupancy; and 8) built environment, materials, and equipment for IPC. ^15^ It is recommended that the implementation of the core components of IPC should be at both the national and facility levels. The first six core components are to be implemented at national level (Ministry of Health in our context) and all the eight should be available at all facility level (at each healthcare facility). The WHO further developed minimum requirements for each core component that stipulates the basic requirements for baseline operational for IPC implementation suitable for low resource settings. ^16^ Facilities should first achieve the minimum requirements, then scale up to full implementation of the core components. However, there is limited tailored guidance for what this means, in practice, at each level.

These core components complement each other. For example, it is suggested that an active IPC program will provide a plan for how other core components can be implemented to improve IPC practices and reduce HAIs. However, for an IPC program to exist, there should also be an enabling environment, reflected in two of the core components (workload, staffing and bed occupancy, and built environment, materials, and equipment). The use of the multimodal improvement strategy (which encompasses system change and infrastructure improvement, education and training of healthcare workers and key players, monitoring and feedback, reminders in the workplace/communications, and culture change, especially in leadership) suggests that a bundle of approaches should be used in conjunction to facilitate optimal implementation.

There are many further challenges to the implementation of the WHO core components of IPC, including shortages of staff (for example, in Malawi, one nurse provides care to 30–80 inpatients per shift, which compromises the care given to every inpatient), ^17^ inadequate IPC training of health care workers (HCWs) and patients/patient guardians, and a lack of IPC resources and supplies (i.e., alcohol-based hand rub, soap, and clean running water). Systemic challenges are often encountered in short-term solutions. For example, as a tentative solution to the shortage of nursing staff, in many African countries, including Malawi, health facilities depend on patient guardians/caregivers to supplement the overburdened workforce. They are directly involved in the patient’s activities of daily living, including bathing, feeding, cooking and transporting them and assisting with rehabilitation exercises. ^18^ ^,^ ^19^ A guardian can be a patient’s relative, friend, or someone who has been assigned to care for the patient during hospitalization by the patient or the patient’s family. Therefore, it is important that guardians be considered and included in the IPC program. Such inclusion could involve providing IPC-related health education for individuals to know what is expected when offering care to patients to prevent avoidable infections. ^17^ ^,^ ^18^ ^,^ ^20^

In Malawi, IPC guidelines have been adapted from a combination of the WHO, Centers for Disease Control and Prevention (CDC), and Infection Control Africa Network (ICAN) guidelines; however, there is no locally adapted plan for implementing the IPC core components. Well-developed guidelines without formal support for their implementation are of limited value. ^12^ ^,^ ^16^ While the WHO has further developed some implementation guidelines for WHO IPC minimum core component requirements, ^16^ these have not yet been contextualised to the Malawian context and there are still gaps in moving this guidance into practice-- adherence to WHO recommendations remains a critical challenge. These gaps were evident in the 2019 Global Survey on Infection Prevention and Control reports. Low-income countries scored at a “basic” level of IPC implementation on average, while notably having limited implementation of IPC guidelines, training and education, monitoring, audit, feedback, and HAI surveillance. ^21^ This raises the question of what factors lead to low adherence to the WHO recommendations in healthcare facilities and what can be done to improve IPC practices and reduce HAIs. There is a dearth of research on IPC implementation, particularly in low-income settings, to help explore how patient safety and quality of care can be improved. In Malawi, there is inconsistent adherence to IPC practices exacerbated by lack of IPC operationalisation guidance, shortages in IPC resources and a lack of training for staff. Responding to these gaps, as the key outcome of this study, we will co-develop locally adapted implementation strategies—interventions that are specifically in place to support the implementation of evidence-based practices—to support the routine use of the WHO Core Components of IPC at the facility level in Malawi.

## Specific study objectives

1. To understand the current landscape of IPC in Malawi, including three acute healthcare facilities, using a mixed-methods situational analysis.
2. To identify potential barriers and enablers of the implementation of the WHO Core Components of IPC at the facility level in Malawi.
3. To use participatory methods to, co-design, with IPC stakeholders context-specific strategies to support the implementation of the WHO Core Components of IPC for use within acute healthcare facilities in Malawi.

## Methods

### Study design

This formative study will have two components. The first is a mixed-methods situational analysis, drawing extensively on qualitative approaches to understand how IPC is currently being conducted at our three study sites. This situational analysis will also include the exploration of potential barriers and enablers of IPC practices. Second, we will conduct a participatory workshop in which key findings from the situational analysis will be shared, after which we will co-design implementation strategies with participating stakeholders to support and improve IPC practices. In line with the best practice guidance for designing and evaluating complex interventions, ^22^ in addition to designing context-appropriate implementation strategies, this formative phase will further provide a basis for building a relational foundation with stakeholders to support the use and evaluation of these implementation strategies in a subsequent study.

The Exploration, Preparation, Implementation and Sustainment (EPIS) framework will be used to guide the overall study design and implementation process. This formative phase aligns with the “Exploration” and “Preparation” stages, focusing on understanding the local context, identifying barriers and facilitators of IPC implementation, facilitating stakeholder engagement, and co-developing feasible and acceptable implementation strategies. ^23^

### Study period

Primary data collection will take place over a period of one year ( Table 1), from April 2023 to March 2024, in Blantyre, Chikwawa, and Zomba. Table 1 below highlights key activities undertaken throughout the study with an emphasis on data collection.

**Table 1.** Study Timeline.

	Months in numbers											
	1	2	3	4	5	6	7	8	9	10	11	12
Study preparation (ethics application, training of research assistants, piloting of tools)												
***Situational analysis***												
Desk/Documentary Review												
IPCAF Survey												
Participant and non-participant observation												
Qualitative data collection (Interviews and FGDs)												
Data Analysis												
***Participatory component***												
Co-design workshop												
Dissemination												

### Study sites

Primary data will be collected from the medical and surgical wards in three health facilities in Malawi: Queen Elizabeth Central Hospital (QECH), Zomba Central Hospital (ZCH) and Chikwawa District Hospital (CDH). These hospitals were purposively selected as they represent secondary and tertiary levels of healthcare facilities, and are teaching, central, and district hospitals, respectively.

QECH provides specialized medical care, and it is the largest urban government teaching and tertiary level hospital in Blantyre, which has a capacity of approximately 1350 beds, but frequently operates above its capacity. ^24^ Located in the Southern region of Malawi, ZCH is a tertiary hospital, although it also works as a secondary level hospital for the Zomba district. ZCH has a capacity of 620 beds but also operates above its capacity. The CDH is a secondary level hospital with a capacity of 250 beds and is situated in the Chikwawa District in southern Malawi.

### Study populations

The study population will include healthcare workers, housekeeping staff, patients, and guardians responsible for carrying out IPC practices in medical and surgical wards, and the leadership/managerial staff who support IPC practices at each site. We will also include public health practitioners, researchers, and other stakeholders from the Ministry of Health, especially from the Quality Management Directorate, as key policy holders for IPC implementation, non-governmental organizations, and funding bodies involved in IPC.

### Sample size and participant recruitment

We estimate that 160 people will be recruited for this situational analysis. As indicated in Table 2, in this first study component, we anticipate including 40 participants in interviews (30 semi-structured interviews with health workers and auxiliary staff, 10 key informant interviews), 60 participants across six focus group discussions (FGDs), and 60 participants in observation. In the second (participatory) component of the study, we will select participants from the situational analysis to partake in the participatory workshop.

**Table 2.** Sample size.

Data collection method	Type of participants	No. participants per facility	Total number of participants
*Situational analysis*			
Participant Observation	Healthcare workers	20	60
Interviews	Healthcare workers	10	30
	IPC stakeholders external to the hospital facilities		10
Focus group discussions *	Cleaners	10	30
	Guardians	10	30
*Participatory research*			
Implementation strategy co-design workshop	Study team	10	42
	National IPC focal persons	2	
	Infection prevention and control Association - Malawi	1	
	Public health Institute of Malawi	1	
	ZCH HCWs **	10	
	QECH HCWs **	10	
	Cleaners	4	
	Patient guardians	4	

* Six to be held in total, one with cleaners and one with guardians per facility, each with approximately 10 participants.

** The group of HCWs will include the representatives from the study wards, facility IPC focal person, the quality improvement focal person and hospital management.

We anticipate recruiting 30 health workers and auxiliary staff for semi-structured interviews—10 per facility. We will sample purposively to ensure maximum variation in terms of age, sex, cadre (e.g. clinicians, IPC focal persons, department management) and years of work experience. Further, the sample sizes in this study are consistent with recommendations in qualitative research. Data collection will occur until theoretical saturation is reached. Saturation will be defined as the point at which no new insights are being found. For interviews, data saturation is usually reached with 12–30 interviews. Therefore, we anticipate that interviewing 30 staff members will be sufficient to reach saturation. Prospective interviewees will be briefed on the study by the study team during a convenient time (e.g. before the morning handover and lunchtimes). After the briefing, information leaflets will be distributed to prospective participants, who will be given at least 24 hours to read through the leaflet and determine whether they would like to participate. The study team will revisit the individual departments/units at least 24 hours after the initial briefing to ask if any individuals are interested in participating. Those individuals will then self-select into the study and will undergo a full informed consent process prior to any data collection.

For key informants, we have mapped out the prospective stakeholders we would like to engage in this study, give their direct role in IPC programming. We have identified 10 IPC stakeholders, including people from the Quality Management Directorate within the Ministry of Health, and representatives of IPC government partners (WHO, Public Health Institute of Malawi, Infection Prevention and Control Association of Malawi). Study information leaflets will be sent to prospective key informants either via email or delivered by the study team in person to offices/places of work. The study team will send a follow-up email, phone call, or visit to ask if any individuals are interested in participating. Again, those individuals will self-select into the study and undergo a full informed consent process prior to being interviewed.

For FGDs, these will be carried out with patient guardians and with hospital cleaners. A size of 6–10 participants is recommended to facilitate meaningful interaction and gain sufficient insights on the phenomena of interest. ^25^ ^,^ ^26^ Overall, we anticipate up to 10 participants per FGD, so have suggested a maximum of 60 participants across these. We aim to conduct six FGDs in total (one with patient guardians and one with cleaning staff in each facility). Based on the size of the medical and surgical units across the facilities and with the desire to cause minimal disruption, we believe that two FGDs per facility will be sufficient for this formative research. Eligible patient guardians will include those who have stayed in the medical or surgical wards for more than two days and are not supporting a critically ill patient to ensure attending a FGD would not interrupt care to their ward. They will be identified in consultation with the healthcare staff. Prospective participants will be given a study information leaflet in the local language and at least 24 hours to consider their participation in the study. These patient guardians will be divided into groups of males and females. Participants who agree will then undergo a written informed consent process. For participants that are not literate, an impartial witness will observe the consent process to ensure that the participant information sheet has been reviewed thoroughly, that the participant has had the opportunity to ask questions, and that any questions have been answered fully. The participant will sign the information sheet with a thumbprint or “X” and the witness will provide their name and signature.

To recruit cleaning staff, the study team will circulate a leaflet about the study and will also post information on hospital noticeboards. After a week or more of study materials being circulated, study staff will approach cleaners and ask them if they wish to participate in the study. All cleaners will undergo a written informed consent process prior to participating in an FGD.

We have planned 60 health worker observations: 20 health workers per site—five per unit (one surgical and medical unit, each divided into male and female units, for a total of four units per facility). As there are roughly 22 health workers on each ward performing the relevant IPC practices we are interested in observing, we have anticipated that observing five health workers per unit will give an adequate indication of how these IPC practices are done.

### Data collection

We will use five different data collection approaches within the situational analysis: 1. A desk/document review; 2. Infection Prevention and Control Assessment Framework (IPCAF) survey; 3. participant and non-participant observation; 4. interviews (semi-structured interviews and key informant interviews); and 5. FGDs. We will use one additional method (a participatory workshop) in the second part of the study. These tools are described below and can be accessed at: https://doi.org/10.5281/zenodo.15202999.

### Situational analysis

***Desk/Document review***

We will conduct an extensive desk/documentary review to identify and review existing IPC and water sanitation and hygiene (WASH) guidelines, policies, standard operating procedures, and protocols for the prevention of HAI at the facility and national levels. Grey literature on IPC will be obtained from the QECH, ZCH, CDH, and Malawi Ministry of Health archives. If archival material is not accessible, we will ask permission to access the material from the relevant authorizing personnel and seek consent, where necessary, for its use as formal research data. To guide desk review and document analysis, a data extraction tool was developed. The tool was based on the study objectives, WHO core components of IPC and national IPC/WASH guidelines.

***Infection Prevention and Control Assessment Framework Survey***

At each study site, an electronic survey will be conducted in the study facilities using the WHO IPCAF tool which is freely available for use by health facilities. The IPCAF is a self-administered, structured tool that will be conducted by the facility IPC focal person with the support of the study team. The IPCAF evaluates the current IPC situation in each facility, based on the eight WHO Core Components of IPC: programming; guidelines; education and training; HAI surveillance; the existence of the multimodal improvement strategy; monitoring and audit and feedback; workload, staffing and bed occupancy; and the built environment. The tool has indicators that are scored up to 100 points per component for a possible total of 800 ( Table 3). It then facilitates the determination of the level of IPC maturity in each domain: inadequate, basic, intermediate, and advanced). The study implementation lead (DN) will facilitate contact with hospital directors from each health facility for permission to use the tool and arrange training on the IPCAF tool. Thereafter, an appropriate date and time will be determined for the assessment. Overall, facility IPCAF scores can be used as indicators of the extent of IPC implementation, highlighting clearly where gaps exist insights that will be used to inform the data collection instruments for interviews, FGDs, and observations.

**Table 3.** IPCAF scoring interpretation.

Score	Level	Interpretation
0–200	Inadequate	IPC CC implementation is deficient. Significant improvement is required.
201–400	Basic	Some aspects of the IPC CCs are in place but not sufficiently implemented. Further improvement is required.
401–600	Intermediate	Most aspects of IPC CC are appropriately implemented. Continue to improve the scope and quality of implementation and focus on the development of long-term plans to sustain and further promote the existing IPC program.
601–800	Advanced	The IPC CCs are fully implemented according to the WHO recommendations and appropriate to the needs of your facility.

### Observation

Observations of IPC practices will be conducted to produce data on what people do (as opposed to what they say they do). During periods of participant (of people) and non-participant observation (of environments and infrastructure rather than behaviours), we are interested in understanding IPC practices, especially those for preventing HAI embedded within daily work routines and interactions between persons.

***Participant observation***

Observations of healthcare workers will be conducted in medical and surgical units across the three sites. We will use a tool which we have developed after review of literature from the CDC, Ministry of Health IPC guidelines ^27^ and assessment tools, Infection Control Africa Network and WHO Indicators are subdivided into standard IPC practices that should be followed when performing urinary catheterization, intravenous cannulation, or wound dressing procedures, specifically those around aseptic technique. For example, performing hand hygiene before aseptic procedure; using non-touch technique when inserting a catheter. Individual HCWs will be observed performing the procedures on five different patients in a specific period. The observations will be conducted by research assistants with IPC knowledge and skills so that they can determine if what was carried out meets the expected standard of care. These research assistants will be integrated into the study wards and maintain a neutral attitude so as not to affect the routine practices. Observing the HCW performing the same procedure a few times and integrating the research assistants in the wards will help reduce the Hawthorne effect, which may result in changed behaviour due to the knowledge of being observed. ^28^ ^,^ ^29^ Data from participant observation will be collected by the observer using a standardized online form built into the Research Electronic Data Capture (REDCap) application.

***Non-participant observation***

Given our wide interest in HAIs and the IPC landscape and environment, our attention will not only be on staff, patients and guardians, but also on the setting. Non-participant observation) will be conducted to determine availability of IPC infrastructure and necessary resources (running clean water, soap, alcohol-based hand rub, mopping buckets), daily practices (ward rounds, cleaning schedules, health education for patients), interactions (between healthcare workers and patients, guardians and cleaners, visitors and guards) and the flow of work (shifts, daily routines). Trained members of the study team will spend time in the medical and surgical units in each facility across five days. The study team will record their observations in field notes.

### Interviews

***Semi-structured interviews***

We will conduct semi-structured interviews with health workers to establish their knowledge of IPC, the current IPC landscape within the study sites and their role in the implementation of the eight IPC core components. Interview guides will be based on national IPC guidelines, eight IPC core components and the IPCAF survey results. Aligned with the EPIS framework, we also aim to better understand contextual factors, especially those of the “inner context” that impact upon IPC implementation.

Research assistants with training and expertise in qualitative data collection, IPC and the Malawian health system will conduct interviews in both Chichewa and English based on the participant’s preference. These will take place within office/hospital spaces, or if no private space is available on site, an alternative private location will be selected. They are expected to take 30–60 minutes.

***Key informant interviews***

Key informant interviews will be conducted to understand the upstream factors (macro-level contextual factors mapping to the “outer context” in the EPIS framework) that shape IPC practices in Malawi, including research related to HAIs, national policy and guideline development, IPC training, governance and coordination of IPC activities, financing and resource allocation and support given to facilities for implementation of IPC practices. Key informant interview guides will be informed by the existing academic and grey literature on IPC from the desk/document review and observational data. Ideally, face-to-face interviews will be conducted by DN and TOB in English; however, online (on Zoom or Teams) or telephone will be offered if necessary. They are expected to take 60–90 min.

### Focus Group Discussions

We will conduct FGDs with (i) patient guardians within the medical and surgical units and (ii) housekeeping staff working within the sites. The purpose of conducting patient guardian FGDs is to determine their understanding of IPC and orientation around IPC while they have been at the hospital, including how they think IPC may improve. FGDs with cleaners will explore their perspectives on IPC, the role that they play in IPC and their understanding of any IPC training they have received.

Each FGD will last between 60–90 minutes and will take place in a quiet location (such as a meeting area inside or outside within the grounds of the hospital). Trained research assistants will work together, with one facilitating the FGD whilst another takes notes.

### Participatory component

***Stakeholder consultation and design workshop***

Co-designing of the implementation strategies will involve a workshop with a selection of key stakeholders who participated in the situational analysis (facility-based healthcare workers and auxiliary staff, IPC key informants, and community members/patient guardians) alongside research staff. Participants will be identified during situational analysis. The workshop will be conducted over a three-day period and will include approximately 42 people. In participatory workshops, findings from the situational analysis will be presented for initial reflection and discussion. The most pressing problems that will emerge from this discussion will be revisited in smaller groups. There, root cause analysis will be carried out using problem trees to identify the major causes and contributing factors to these larger problems. Participants will then rank the root causes to address using a problem prioritisation matrix, which will be based on feasibility, affordability and impact. Following this exercise, participants will reflect against the root causes that will be deemed to be the most feasible, affordable and impactful to address, and from these, a group brainstorming activity will be carried out in which possible activities to address these root causes could be undertaken. These, too, will be prioritised on the basis of feasibility, affordability and impact. Overlapping activities will be synthesised into wider implementation strategies that could accomplish them, leading to the creation of the implementation strategies. This participatory approach will ensure that the selected intervention and implementation strategies are contextually relevant, feasible and supported by key stakeholders, thereby enhancing acceptability and likelihood of implementation.

Data will be collected during this workshop through observations and note-taking of interactions and dynamics between participants. Photographs of the process will also be taken.

### Data analysis

Descriptive statistics and framework analysis will be used to identify patterns and key insights relevant to the study’s objectives.

***Desk review***

The selected documents will be appraised against the WHO core component guidance to identify the gaps and strengths that exist in the guidelines/IPC documents at the facility level. We will then compare and aggregate these findings with data from other sources.

***IPCAF survey and participant observation***

The IPCAF data will be analysed in Microsoft Excel using descriptive statistics. The IPCAF scores will be summarized using median and interquartile ranges. The mean scores will also be calculated. Data from the HCWs’ observation checklists will be analysed in Excel and presented as data summaries, reports, and graphs.

***Interviews and FGDs***

Qualitative data in the form of field notes and interview and FGD transcripts will be uploaded into NVivo (Version 12). https://www.lumivero.com/ for organising the data during coding. Recorded data will be transcribed and translated. Anonymized transcripts will be analysed using a combined deductive framework analysis, followed by an inductive thematic analysis within each component of the framework. As the coding advances within each framework component, higher order codes will be developed to explain and theorize the patterns and themes in the data. Coding reliability will be checked done by double coding 20% of the transcript noting coder agreement and resolving disagreements. This process will continue with another 20% of the transcripts if there is a high level of disagreement initially, until it is clear that coding is consistent between researchers. Findings and interpretations, including coded transcripts, fieldnote summaries and written analyses, will be discussed with the wider IPC-Implement research team. Within each interview or FGD, research assistants are trained to paraphrase responses to ensure they have accurately captured the participant’s meaning, for an immediate check-in during data collection. Member checking of the qualitative analysis will also be done during the participatory workshop, as we will share our analysed findings for reflection across participants to determine, “if we got it right” and to increase our interpretive validity. These reflections will then be used to generate implementation strategies to improve IPC practices in the CDH, ZCH, and QECH.

### Cross-site analysis

The study team will conduct regular reflection meetings to discuss emerging data as the research progresses, inviting representatives from the Ministry of Health to join these meetings when available. Case classifications were used to denote characteristics of each transcript, including which facility these are from. Using the coding comparison functions in NVivo as an initial first step, we will determine the similarities and differences across each facility. By comparing the analyses, we aim to identify common themes and connections between sites, as well as points of departure and differences.

### Data management

Data will be managed through infrastructure set up within the Malawi-Liverpool-Wellcome (MLW) Research Programme. Observational data and IPCAF survey data will be stored on the MLW REDCap server, anonymized, and transferred to a Microsoft Excel spreadsheet for analysis.

IPCAF, and observations data will be collected using tablets and will be uploaded to REDCap. Interview and FGD recordings, transcripts and field notes will be kept in a folder on a password protected computer. The data will be backed up to the MLW’s secure cloud storage. The full anonymization process will occur following peer-reviewed publication, but all contact details collected from participants throughout the study will be destroyed at the end of the study follow-up for data cleaning and query resolution. Once transcripts have been generated and quality checked from the audio files, the researcher will delete the audio files. Transcripts will have participants’ identifying information redacted.

All raw data will be stored in password-protected files/folders, and second-order summaries of field notes, interview transcripts, photos, and other field documents will be uploaded to a data repository (with participant consent). Details on how to access the data will be published with each study publication, and access will be granted based on a case-by-case request and approval. Aggregated IPC indicators (e.g. IPCAF scores) and de-identified observational summaries will be shared in Excel upon reasonable request, subject to ethical approval. Qualitative data will not be shared in full, however, redacted extracts may be shared after analysis on request to the study team.

The data and paper-based records of this project will be destroyed after five years. All devices and paper-based tools containing data will be securely stored in offices at the MLW Data Office and in a locked data repository room for long-term storage. The MLW Data Office will perform daily data backups, with an offsite backup done weekly.

## Ethics and consent

Ethical approvals to conduct the study were obtained from the Kamuzu University of Health Sciences Research Ethics Committee (COMREC P.02/23/3993 on 17 May 2023) and the Liverpool School of Tropical Medicine Research Ethics Committee (LSTM REC 23–007 on 2 June 2023). Before commencing research activities, we will approach hospital management, heads of department and the HCWs from the medical and surgical departments to explain the objectives of the study and seek permission to work in the proposed areas. This study will adhere to the Declaration of Helsinki.

At the time of enrolment, written informed consent forms and information sheets on study participation will be provided for each adult participant (over the age of majority according to local regulations in Malawi this is 18 years of age). Where applicable, the consent forms will include consent for audio recordings, the use of direct quotes, and photography (within the participatory workshop only), which participants may opt out of but still maintain their participation.

The formative research will be observational and thus is not expected to pose harm to participants, barring interruptions, or minor inconveniences from participating in research activities. However, we will only conduct research activities at appropriate times as directed by participants. We are conscious that we may take up people’s time and will therefore ensure that we arrange data collection at times suitable to the participant, respecting when there may be cancellations or postponements. Participants travelling to workshops will be reimbursed for their transport and accommodation.

During the research process, it is possible to uncover poor IPC practices and/or poor clinical practices that need to be resolved. During such times, we will ensure that such issues are first discussed with the principal investigator and then with the relevant individuals in and heads of the department. For those being observed, it is possible for individuals to feel scrutinized or uncomfortable. We will stress that they will not be identifiable from any notes that we have taken. If individuals voice concerns about being observed, they will not be observed.

The in-depth nature of data collection methods, particularly participant observation, safeguarding participants privacy is a priority. We will implement strict measures to ensure their privacy is respected. All interviews, and FGDs will be conducted in a private space where conversations cannot be overheard. Where participant observation takes place in public spaces, it will be made very clear to participants at the outset that they can make informed choices as to when to share information. Study researchers will ensure that participants are aware that only the research team will have access to raw data (including both raw data and data uploaded to REDCap). Any data shared beyond the group will be subject to approval by the study team on a case-by-case basis.

We will ensure that participants fully understand their participation in the study is voluntary and that they will not face any consequences should they choose not to respond to some questions, refuse to participate, or withdraw from the study at any point. More broadly, we will ensure that participants understand that this study aims to contribute to improved policies and guidelines for IPC that will benefit people in Malawi.

All participants will be assigned pseudonyms in publications and other research outputs from this study. In the case of public health practitioners, policymakers, and other stakeholders—individuals with more unique and potentially identifiable roles—it will be made clear during the consent process that there may be limits to their anonymity in research output. We will also offer them the option of not using direct quotations from the interviews.

## Dissemination of the results

Dissemination of situational analysis is, in part, built within this study through a participatory workshop. However, findings will be shared immediately after the situational analysis through feedback workshops with staff working in the medical and surgical departments who are not part of the workshop. After the participatory workshop we will then hold wider dissemination meetings of study findings with the CDH, QECH, and ZCH senior management teams. The results will also be presented through an ongoing dialogue with the Quality Management Directorate technical working group, including zonal officers. We will also share our findings with the Kamuzu University of Health Sciences Research Ethics Committee, including other academics, on their research dissemination day. The results from this study will be further disseminated to other stakeholders, including additional Ministry of Health Malawi representatives, the College of Medicine Research Ethics Committee and the Liverpool School of Tropical Medicine Research Ethics Committee as per reporting requirements. We will also present findings at national and international conferences and aim to publish them in academic journals.

## Expected findings

The key outputs from this work will be: 1. Evidence of IPC practices implementations in three hospitals in southern Malawi, including an improved understanding of key bottlenecks and opportunities for strengthening IPC practices; and 2. co-designed implementation strategies to enable routine use of the IPC Core Components. These findings will be reported in terms of baseline IPC practice compliance percentage and IPCAF scores, alongside rich qualitative descriptions of facilitators and barriers. Detailed implementation strategies will be described, alongside tools to affect their use (e.g. training materials, revised guidelines). We will have insights into who will use and champion these implementation strategies, where and how they should be used, and for how long. We will then build on these outputs, supporting and evaluating these implementation strategies beginning in 2025.

## Data Availability

No data associated with this article. Data collection tools. https://doi.org/10.5281/zenodo.15202999. ^30^ This project contains the following extended data:
• Document review guide • Infection prevention and control assessment framework tool • Healthcare observation tool • Participant information leaflet for patient guardians and cleaning staff Data available under CC By 4.0 Creative Commons Attribution 4.0 International.
