# Supplementary material for: Designing implementation strategies for improving infection prevention and control in acute healthcare facilities in Malawi: A formative study protocol
Source: Wellcome Open Res. 2026 Apr 9;10:223. Originally published 2025 Apr 28. [Version 4] doi: 10.12688/wellcomeopenres.24040.4 (PMC13000401; doi:10.12688/wellcomeopenres.24040.4)
Supplement: Peer review history

# Reviewer report: Reviewer response for version 4

**DOI:** 10.21956/wellcomeopenres.28812.r152967

**Published:** 2026-04-16

**Authors:** Eva Cappelli

**Affiliations:** 1 Universita degli Studi di Verona, Verona, Veneto, Italy

Dear Authors,

 Thank you for your thoughtful and thorough responses to my comments on your protocol entitled "Dorica Ng'ambi – Malawi-Liverpool-Wellcome Trust Clinical Research Programme, Blantyre, Malawi".

 I appreciate the clarity and detail you have provided in your replies. Specifically:

 The explicit use of the EPIS framework (Exploration, Preparation, Implementation, Sustainment) now provides a solid implementation science grounding to the study.

 The additional detail on the participatory workshop process – including problem trees, prioritisation matrices based on feasibility, affordability and impact, and the synthesis of overlapping activities into implementation strategies – greatly enhances methodological transparency and replicability.

 Your acknowledgment of staff shortages and the integration of feasibility considerations into the participatory design, ensuring that proposed strategies fit within existing roles and supervisory structures, demonstrates a realistic and context-sensitive approach.

 The explicit mention of mitigation strategies for the Hawthorne effect under data collection strengthens the rigour of the observation methods.

 The clarification on future data accessibility, including which datasets will be shared and in what formats, as well as the curation of qualitative excerpts to protect confidentiality, aligns well with open science principles.

 Overall, you have done an excellent job addressing all the points raised. For my opinion, the protocol is now methodologically robust, clearly described, and well-suited to the resource-constrained setting it aims to serve.

 My recommendation: the manuscript is ready for indexing.

Is the study design appropriate for the research question?

Yes

Is the rationale for, and objectives of, the study clearly described?

Yes

Are sufficient details of the methods provided to allow replication by others?

Partly

Are the datasets clearly presented in a useable and accessible format?

Not applicable

Reviewer Expertise:

Infection prevention and control; nursing leadership and management; qualitative research, with expertise in implementation processes and health systems in resource-constrained settings.

I confirm that I have read this submission and believe that I have an appropriate level of expertise to confirm that it is of an acceptable scientific standard.

# Reviewer report: Reviewer response for version 4

**DOI:** 10.21956/wellcomeopenres.28812.r152969

**Published:** 2026-04-15

**Authors:** Jennifer JP Collins

**Affiliations:** 1 University of the West of Scotland, Paisley, Scotland, UK

Thank you for addressing my comments. I have no further comments to make.

Is the study design appropriate for the research question?

Yes

Is the rationale for, and objectives of, the study clearly described?

Yes

Are sufficient details of the methods provided to allow replication by others?

Yes

Are the datasets clearly presented in a useable and accessible format?

Yes

Reviewer Expertise:

NA

I confirm that I have read this submission and believe that I have an appropriate level of expertise to confirm that it is of an acceptable scientific standard.

# Reviewer report: Reviewer response for version 3

**DOI:** 10.21956/wellcomeopenres.28214.r144787

**Published:** 2026-03-18

**Authors:** Nehad J Ahmed

**Affiliations:** 1 Prince Sattam Bin Abdulaziz University, Al Kharj, Saudi Arabia

The authors responded to almost all comments:

 Minor issues still present

1. No formal primary/secondary outcomes
2. Coding reliability description still brief
3. Dissemination plan not very detailed

However, none of these are major methodological flaws.

Is the study design appropriate for the research question?

Yes

Is the rationale for, and objectives of, the study clearly described?

Yes

Are sufficient details of the methods provided to allow replication by others?

Partly

Are the datasets clearly presented in a useable and accessible format?

Yes

Reviewer Expertise:

NA

I confirm that I have read this submission and believe that I have an appropriate level of expertise to confirm that it is of an acceptable scientific standard.

# Reviewer report: Reviewer response for version 3

**DOI:** 10.21956/wellcomeopenres.28214.r145012

**Published:** 2026-02-16

**Authors:** Jennifer JP Collins

**Affiliations:** 1 University of the West of Scotland, Paisley, Scotland, UK

Thank you for the opportunity to review this pertinent research, which I believe will bring a clear added value to the existing scientific literature on infection, prevention and control implementation

**Comment 1. **It would be useful to more clearly state in the introduction when discussing facility level vs national as this is not explicit to the reader at present. It may also be helpful to stress the difference between the minimum requirements and the core components when introducing these.

**Comment 2. **It is recommended to include relevant WASH documents within the desk review, as several pertinent guidance on this overlap and include components on Infection, prevention and control at the facility level (such as the WASHFIT guideline). This is particularly important as the study includes considerations for the hospital environment.

**Comment 3. **The methods mention using IPCAF in surgical and medical departments. As this is a facility wide tool it is not clear what is meant by this, and how this would be conducted. It is important to clarify why these departments alone are mentioned (in relation to the IPCAF). It would also help to understand if this suggests a need for tool adaption if only being used in departments, given the questions and indicators focus on the facility level. This should be addressed to ensure scientific rigor.

**Comment 4. **It would be important to consider measures to mitigate observer bias within the observation of IPC practices, or the consideration for this within the data analysis. These should be included within the methodology and/or data analysis section.

**Comment 5. **The inclusion of cleaners within the study is pertinent and should bring an important under reported perspective to the study.

**Comment 6. **As the research focuses on implementation it would be useful to draw on frameworks from Implementation Science and to structure the components within these.

 Reference: World Health Organization & United Nations Children’s Fund (UNICEF). (2022). Water and sanitation for health facility improvement tool (WASH FIT): A practical guide for improving quality of care through water, sanitation and hygiene in health care facilities (2nd ed.). World Health Organization.

Is the study design appropriate for the research question?

Yes

Is the rationale for, and objectives of, the study clearly described?

Yes

Are sufficient details of the methods provided to allow replication by others?

Yes

Are the datasets clearly presented in a useable and accessible format?

Yes

Reviewer Expertise:

NA

I confirm that I have read this submission and believe that I have an appropriate level of expertise to confirm that it is of an acceptable scientific standard, however I have significant reservations, as outlined above.

# Response

**Published:** 2026-02-27

**Authors:** Dorica Ng'ambi

**Affiliations:** Malawi-Liverpool-Wellcome Trust Clinical Research Programme, Blantyre, Southern Region, Malawi

Thank you for the opportunity to review this pertinent research, which I believe will bring a clear added value to the existing scientific literature on infection, prevention and control implementation

**Comment 1. **It would be useful to more clearly state in the introduction when discussing facility level vs national as this is not explicit to the reader at present. It may also be helpful to stress the difference between the minimum requirements and the core components when introducing these.

**Response 1.** Thank you for this comment, this has been addressed in the introduction

**Comment 2. **It is recommended to include relevant WASH documents within the desk review, as several pertinent guidance on this overlap and include components on Infection, prevention and control at the facility level (such as the WASHFIT guideline). This is particularly important as the study includes considerations for the hospital environment.

**Response 2.** Thank you for your observation. This has been included under the desk/document review section. It was an oversight, as already in Malawi the IPC guidelines includes the WASH component.

**Comment 3. **The methods mention using IPCAF in surgical and medical departments. As this is a facility wide tool it is not clear what is meant by this, and how this would be conducted. It is important to clarify why these departments alone are mentioned (in relation to the IPCAF). It would also help to understand if this suggests a need for tool adaption if only being used in departments, given the questions and indicators focus on the facility level. This should be addressed to ensure scientific rigor.

**Response 3.** Thank you for noting this mistake, it has been corrected the IPCAF survey will be conducted for the facility not in the previously mentioned departments.

**Comment 4. **It would be important to consider measures to mitigate observer bias within the observation of IPC practices, or the consideration for this within the data analysis. These should be included within the methodology and/or data analysis section.

**Response 4.** Thank you for this comment. This has been addressed under data collection

**Comment 5. **The inclusion of cleaners within the study is pertinent and should bring an important under reported perspective to the study.

**Response 5.** Thank you.

**Comment 6. **As the research focuses on implementation it would be useful to draw on frameworks from Implementation Science and to structure the components within these.

**Response 6.** Thank you for this comment. We have included the Exploration, Preparation, Implementation and Sustainment framework as its overall phased approach aligns very well with our study design. This formative phase aligns with the “exploration” and “preparation” phases of EPIS, and much of the qualitative enquiry we will carry out maps to better understanding “inner” and “outer” contextual factors. We have made this more clear in the manuscript.

# Reviewer report: Reviewer response for version 3

**DOI:** 10.21956/wellcomeopenres.28214.r145013

**Published:** 2026-01-23

**Authors:** Eva Cappelli

**Affiliations:** 1 Universita degli Studi di Verona, Verona, Veneto, Italy

This is a timely and ambitious study addressing a critical gap in the implementation of Infection Prevention and Control practices in resource-constrained healthcare settings. The following comments aim to strengthen the clarity and methodological rigour of an otherwise strong protocol.

 - The methods are generally well described and reflect a robust and well-integrated mixed-methods design. The use of multiple data sources (IPCAF, observations, interviews, FGDs, and a participatory workshop) represents a major strength of the protocol. However, some elements would benefit from further clarification to fully support replication. In particular, the protocol does not explicitly state the implementation science framework guiding the identification and interpretation of barriers and enablers, which may limit analytical transparency and comparability across studies (e.g. Consolidated Framework for Implementation Research, Theoretical Domains Framework, Capability–Opportunity–Motivation–Behaviour).

 In addition, the process by which findings from the situational analysis will be translated into specific and prioritised implementation strategies during the participatory workshop could be described in more detail (e.g. criteria for prioritisation, decision-making or consensus processes).

 Given the well-documented staff shortages in the study context, further clarification on how the proposed implementation strategies will account for existing workload constraints and long-term sustainability would also be valuable. Finally, although observation methods are clearly outlined, the manuscript could more explicitly address how potential observer effects (e.g. Hawthorne effect) will be mitigated. Addressing these points would strengthen methodological clarity, feasibility, and reproducibility.

 - As this is a study protocol, datasets are not yet available; however, data management and anonymisation procedures are clearly described and represent a strength. Greater clarity would nonetheless be beneficial regarding future data accessibility and reuse, for example by specifying which datasets (e.g. aggregated IPCAF scores or de-identified observation summaries) will be shared, in what formats, and under what access conditions. Brief clarification on how qualitative data excerpts will be curated to protect confidentiality would further strengthen transparency and alignment with open science practices.

Is the study design appropriate for the research question?

Yes

Is the rationale for, and objectives of, the study clearly described?

Yes

Are sufficient details of the methods provided to allow replication by others?

Partly

Are the datasets clearly presented in a useable and accessible format?

Not applicable

Reviewer Expertise:

Infection prevention and control; nursing leadership and management; qualitative research, with expertise in implementation processes and health systems in resource-constrained settings.

I confirm that I have read this submission and believe that I have an appropriate level of expertise to confirm that it is of an acceptable scientific standard, however I have significant reservations, as outlined above.

## References

[1] : The Consolidated Framework for Implementation Research (CFIR) User Guide: a five-step guide for conducting implementation research using the framework. *Implementation Science* .2025;20(1) : 10.1186/s13012-025-01450-7 10.1186/s13012-025-01450-7 40819067 PMC12357348

[2] : Head Nurse Leadership: Facilitators and Barriers to Adherence to Infection Prevention and Control Programs—A Qualitative Study Protocol. *Nursing Reports* .2024;14(3) : 10.3390/nursrep14030138 1849-1858 10.3390/nursrep14030138 PMC1134803839189268

[3] : The Nursing Organisational Context and the Surgical Site Infections: A Structural Equational Model. *Nursing Open* .2025;12(11) : 10.1002/nop2.70372 10.1002/nop2.70372 PMC1264381941286584

# Response

**Published:** 2026-02-27

**Authors:** Dorica Ng'ambi

**Affiliations:** Malawi-Liverpool-Wellcome Trust Clinical Research Programme, Blantyre, Southern Region, Malawi

This is a timely and ambitious study addressing a critical gap in the implementation of Infection Prevention and Control practices in resource-constrained healthcare settings. The following comments aim to strengthen the clarity and methodological rigour of an otherwise strong protocol. Thank you.

**Comment 1. **The methods are generally well described and reflect a robust and well-integrated mixed-methods design. The use of multiple data sources (IPCAF, observations, interviews, FGDs, and a participatory workshop) represents a major strength of the protocol. However, some elements would benefit from further clarification to fully support replication. In particular, the protocol does not explicitly state the implementation science framework guiding the identification and interpretation of barriers and enablers, which may limit analytical transparency and comparability across studies (e.g. Consolidated Framework for Implementation Research, Theoretical Domains Framework, Capability–Opportunity–Motivation–Behaviour).

**Response 1. **Thank you for this comment. We are using the Exploration, Preparation, Implementation and Sustainment (EPIS) framework and have described how this has been used in this phase of the study.

**Comment 2.** In addition, the process by which findings from the situational analysis will be translated into specific and prioritised implementation strategies during the participatory workshop could be described in more detail (e.g. criteria for prioritisation, decision-making or consensus processes).

**Response 2. **We agree and have added more detail here. Specifically, we have indicated that, “ *. In participatory workshops, findings from the situational analysis will be presented for initial reflection and discussion. The most pressing problems that will emerge from these discussion will be revisited in smaller groups. There, root cause analysis will be carried out using problem trees to identify the major causes and contributing factors to these larger problems. Participants will then rank the root causes to address using a problem prioritisation matrix, which will be based on feasibility, affordability and impact. Following this exercise, participants will reflect against the root causes that will be deemed to be the most feasible, affordable and impactful to address, and from these, a group brainstorming activity will be carried out in which possible activities to address these root causes could be undertaken. These, too, will be prioritised on the basis of feasibility, affordability and impact. Overlapping activities will be synthesised into wider implementation strategies that could accomplish them, leading to the creation of the implementation strategies.”*

**Comment 3.** Given the well-documented staff shortages in the study context, further clarification on how the proposed implementation strategies will account for existing workload constraints and long-term sustainability would also be valuable.

**Response 3.** Thank you for this observation. As participatory research will be used to establish the implementation strategies, the well-known staff shortages were taken into consideration by stakeholders, particularly as they reflected around “feasibility” aspects of activities. As such, stakeholders proposed ways in which the proposed implementation strategies could be incorporated into routine work in the facilities, and how newly established responsibilities may fit within existing roles and supervisory structures in the facilities.

**Comment 4.** Finally, although observation methods are clearly outlined, the manuscript could more explicitly address how potential observer effects (e.g. Hawthorne effect) will be mitigated. Addressing these points would strengthen methodological clarity, feasibility, and reproducibility.

**Response 4.** Thank you for this observation, we have addressed the Hawthorne effect under data collection.

**Comment 5.** As this is a study protocol, datasets are not yet available; however, data management and anonymisation procedures are clearly described and represent a strength. Greater clarity would nonetheless be beneficial regarding future data accessibility and reuse, for example by specifying which datasets (e.g. aggregated IPCAF scores or de-identified observation summaries) will be shared, in what formats, and under what access conditions. Brief clarification on how qualitative data excerpts will be curated to protect confidentiality would further strengthen transparency and alignment with open science practices.

**Response 5. **Thank you for this suggestion. We have clarified and added the anticipated data sharing approach.

# Reviewer report: Reviewer response for version 1

**DOI:** 10.21956/wellcomeopenres.26524.r128138

**Published:** 2025-09-02

**Authors:** Nehad J Ahmed

**Affiliations:** 1 Prince Sattam Bin Abdulaziz University, Al Kharj, Saudi Arabia

**Comments**

**Study Objectives**

- Add a sentence or two summarizing existing IPC gaps in Malawi. Furthermore, add a bridging sentence (e.g., "Given these gaps, this study aims to co-design tailored IPC strategies by …………. to improve adherence in Malawi.
- Define the primary and secondary outcomes, even for a formative study, to help frame the analysis and ensure clarity for future implementation phases.

**Methodology  **

-  While the sample size (200 participants) is stated, the rationale for choosing 30 HCWs for interviews, 60 for observations, and 10 per FGD is not explicitly justified. Add a brief justification.
-  Clarify recruitment protocols (Explain strategies to ensure representation across different staff levels, wards, and patient guardian backgrounds).
- Please include a brief description of the data collection tools, their core variables, and concepts measured by each tool.
- Specify approach for informed consent with participants of limited literacy — who will verify understanding, and how?.
- The qualitative analysis plan mentions a combined deductive and inductive approach but lacks detail on coding reliability. Add more data about coding reliability and how discrepancies will be resolved.
- The plan for qualitative analysis (NVivo 12) is sound but does not mention member checking or peer debriefing to enhance credibility.

**Dissemination of the results**

- While various dissemination activities and audiences are mentioned (workshops, meetings, conferences), the plan does not specify timing, frequency, or formats (e.g., reports, policy briefs, plain language summaries) tailored for different stakeholders.

**Expected findings**

- The section presents anticipated outcomes but lacks measurable indicators or metrics to evaluate success.

**General comments **

- A Gantt chart or table summarizing the timeline for each study phase would improve clarity.
- The plain language summary could better emphasize the study’s potential impact on patient outcomes. Reframe the summary to highlight how the co-designed strategies could reduce HAIs and save lives in Malawi.

Is the study design appropriate for the research question?

Yes

Is the rationale for, and objectives of, the study clearly described?

Yes

Are sufficient details of the methods provided to allow replication by others?

Partly

Are the datasets clearly presented in a useable and accessible format?

Yes

Reviewer Expertise:

Clinical Pharmacy

I confirm that I have read this submission and believe that I have an appropriate level of expertise to confirm that it is of an acceptable scientific standard, however I have significant reservations, as outlined above.

# Response

**Published:** 2025-12-11

**Authors:** Dorica Ng'ambi

**Affiliations:** Malawi-Liverpool-Wellcome Trust Clinical Research Programme, Blantyre, Southern Region, Malawi

**Comment 1**: Add a sentence or two summarizing existing IPC gaps in Malawi. Furthermore, add a bridging sentence (e.g., "Given these gaps, this study aims to co-design tailored IPC strategies by …………. to improve adherence in Malawi

**Response 1:**Thank you for this comment. We have added a summary of the existing IPC implementation gaps and have provided references for this. We have added a bridging sentence to demonstrate how these gaps informed the study design.

**Comment 2: **Define the primary and secondary outcomes, even for a formative study, to help frame the analysis and ensure clarity for future implementation phases.

**Response 2: **We have noted the key outcome of this study being the adapted implementation strategies and other outputs. We have reiterated under “expected findings” that we also intend to build the evidence base around IPC implementation. There are no primary and secondary outcomes per se, but we have provided more detail about the outputs we are anticipating from this work within that section.

**Comment 3: **While the sample size (200 participants) is stated, the rationale for choosing 30 HCWs for interviews, 60 for observations, and 10 per FGD is not explicitly justified. Add a brief justification.

**Response 3: **Thank you for this comment. We have added in further justification of sample size within that section.

**Comment 4: **Clarify recruitment protocols (Explain strategies to ensure representation across different staff levels, wards, and patient guardian backgrounds).

**Response 4: **We have moved information about the recruitment procedure within the section on participant sampling, which is now “Participant Sampling and Recruitment”. We have added in more detail for each type of participant where relevant.

**Comment 5: **Please include a brief description of the data collection tools, their core variables, and concepts measured by each tool.

**Response 5: **Thank you—we agree the detail for each instrument can be strengthened and have added relevant detail to each tool as described in the section on data collection. 

**Comment 6: **Specify approach for informed consent with participants of limited literacy — who will verify understanding, and how?

**Response 6: **We have added more detail around participant recruitment and informed consent in the section on sampling. Only patient guardians were not literate, and the process of obtaining consent from these participants is detailed within the description of the focus group discussions.

**Comment 7: **The qualitative analysis plan mentions a combined deductive and inductive approach but lacks detail on coding reliability. Add more data about coding reliability and how discrepancies will be resolved.

**Response 7: **We have included a description of how we ensured inter-coder reliability.

**Comment 8: **The plan for qualitative analysis (NVivo 12) is sound but does not mention member checking or peer debriefing to enhance credibility.

**Response 8: **We agree and have added a sentence on how this will be done as data collection progresses and post-analysis.

**Comment 9: **While various dissemination activities and audiences are mentioned (workshops, meetings, conferences), the plan does not specify timing, frequency, or formats (e.g., reports, policy briefs, plain language summaries) tailored for different stakeholders.

**Response 9: **We have clarified timing and added more detail on the format of dissemination within this section.

**Comment 10: **Expected findings -****** The section presents anticipated outcomes but lacks measurable indicators or metrics to evaluate success.

**Response 10: **We have added further details here but note many of our outputs are not necessarily quantifiable into clear indicators, as they will be exploratory qualitative data and implementation strategies.

**Comment 11: **A Gantt chart or table summarizing the timeline for each study phase would improve clarity.

**Response 11**: Thank you. We have provided a figure to indicate the timing, as we agree showing when we anticipate collecting each type of data will be helpful to understand the iterative nature of the research, as different instruments inform subsequent data collection.

**Comment 12: **The plain language summary could better emphasize the study’s potential impact on patient outcomes. Reframe the summary to highlight how the co-designed strategies could reduce HAIs and save lives in Malawi.

**Response 12: **Thank you, we have added a sentence at the end of this section to address this.
